# Cross-Resistance to Pyrethroids and Neonicotinoids in Malaria Vectors from Vegetable Farms in the Northern Benin

**DOI:** 10.3390/tropicalmed9120305

**Published:** 2024-12-12

**Authors:** Massioudou Koto Yérima Gounou Boukari, Innocent Djègbè, Ghislain T. Tepa-Yotto, Donald Hessou-Djossou, Genevieve Tchigossou, Eric Tossou, Michel Lontsi-Demano, Danahé Adanzounon, Adam Gbankoto, Luc Djogbénou, Rousseau Djouaka

**Affiliations:** 1 Département des Sciences de la Vie et de la Terre, Ecole Normale Supérieure de Natitingou, Natitingou P.O. Box 72, Benin; dowoogateh3d@yahoo.com; 2 Agroecohealth Unit, International Institute of Tropical Agriculture (IITA), 08 Tri-Postal, Cotonou P.O. Box 0932, Benin; g.tchigossou@cgiar.org (G.T.); e.tossou@cgiar.org (E.T.); l.michel@cgiar.org (M.L.-D.); d.adanzounon@cgiar.org (D.A.); r.djouaka@cgiar.org (R.D.); 3 Biorisk Management Facility (BIMAF), International Institute of Tropical Agriculture (IITA-Benin), 08 Tri-Postal, Cotonou P.O. Box 0932, Benin; g.tepa-yotto@cgiar.org; 4 Ecole de Gestion et de Production Végétale et Semencière (EGPVS), Université Nationale d’Agriculture (UNA-Benin), Kétou BP 43, Benin; 5 Laboratory of Experimental Physiology and Pharmacology, Faculty of Sciences and Technology, University of Abomey-Calavi, Cotonou 01 BP 526, Benin; qgoba@yahoo.fr; 6 Regional Institute of Public Health, University of Abomey-Calavi, Ouidah P.O. Box 384, Benin; ldjogbenou22002@yahoo.fr

**Keywords:** *Anopheles gambiae* s.l., resistance, insecticides, vegetable farm, Benin

## Abstract

Agricultural pesticides may play a crucial role in the selection of resistance in field populations of mosquito vectors. This study aimed to determine the susceptibility level of *An. gambiae* s.l. to pyrethroids and neonicotinoids in vegetable farms in northern Benin, in West Africa, and the underlying insecticide resistance mechanisms. A survey on agricultural practices was carried out on 85 market gardeners chosen randomly in Malanville and Parakou. *Anopheles gambiae* s.l. larvae were collected, reared to adult stages, and identified to species level. Susceptibility was tested with impregnated papers (WHO bioassays) or CDC bottles according to the insecticides. Synergists (PBO, DEM, and DEF) were used to screen resistance mechanisms. Allelic frequencies of the *kdr (L1014F)*, *kdr (L1014S)*, *N1575Y*, and *ace-1R G119S* mutations were determined in mosquitoes using Taqman PCR. Fertilizers and pesticides were the agrochemicals most used with a rate of 97.78% and 100%, respectively, in Malanville and Parakou. *Anopheles coluzzii* was the predominant species in Malanville, while *An. gambiae* was the only species found in Parakou. Bioassays revealed a high resistance of *An. gambiae* s.l. to pyrethroids and DDT, while a susceptibility to bendiocarb, pyrimiphos-methyl, malathion, and clothianidin was recorded. Resistance to acetamiprid was suspected in mosquitoes from both localities. A lower resistance level was observed when mosquitoes were pre-treated with synergists, then exposed to insecticides. The *kdr L1014F* mutation was observed in both locations at moderate frequencies (0.50 in Malanville and 0.55 in Parakou). The allelic frequencies of *N1575Y* and *G119S* were low in both study sites. This study confirmed the resistance of *An. gambiae* s.l. to insecticides used in agriculture and public health. It reveals a susceptibility of vectors to bendiocarb, pyrimiphos-methyl, malathion, and clothianidin, thus indicating that these insecticides can be used as an alternative in Benin to control malaria vectors.

## 1. Introduction

In the last decades, malaria vector control using insecticide-treated mosquito nets (ITNs) and indoor residual spraying (IRS) has significantly reduced the spread of *Plasmodium* infection in Africa [1]. Out of the 663 million cases averted in sub-Saharan Africa between 2000 and 2015, ITNs and IRS contributed to 68% and 13% of interventions, respectively [2]. Unfortunately, this level of intervention may become difficult to maintain in the future if the rapid spread of insecticide resistance observed among malaria vectors is not managed effectively [1,3].

In Benin, studies carried out over the last two decades reported the misuse and overuse of agrochemical products leading to the contamination of mosquito larvae breeding sites and the spread of insecticide resistance in malaria vectors [4,5,6,7,8,9]. Furthermore, most insecticides used in agriculture are of the same chemical classes, having the same targets and modes of action as those used for vector control in public health [10,11], thus constituting the resistance selection pressure in mosquito populations.

In order to make these insecticides more effective, the WHO introduced a synergist, piperonyl butoxide (PBO), to increase insecticide efficacy, but the resistance of malaria vectors to insecticides has not been completely eliminated [4]. Piperonyl butoxide (PBO) is an insecticide synergist known to inhibit the activity of cytochrome P450 enzymes [12]. Its ability to inhibit major metabolic resistance enzymes makes it an ideal synergist to enhance the insecticide effect. However, due to the threat posed by insecticide resistance, a new class of insecticides such as neonicotinoids, especially clothianidin, has recently been recommended for public health by the WHO [13]. Clothianidin developed in two formulations by both Sumitomo (solo, under the name SumiShield) and Bayer (as a combination with deltamethrin, under the name Fludora Fusion) was evaluated and prequalified by the WHO in 2017 [14]. These two formulations tested on different species of malaria vectors have shown their effectiveness. Indeed, the mode of action of clothianidin is to target the nicotinic acetylcholine receptor (nAChR) in the insect central nervous system [15,16]. The strict monitoring of the susceptibility of mosquitoes to new molecules is necessary as much as possible in order to avoid the appearance of a widespread resistance [17]. 

The use of this new class of insecticides in Benin could help the National Malaria Control Program to meet its challenge of no longer seeing malaria as a major public health problem by 2030. However, insecticides having the same mode of action as clothianidin, notably acetamiprid, are being used in agriculture in Benin [18,19,20]. Very little data exist on the resistance profile of *Anopheles (An.) gambiae sensu lato* (s.l.) to neonicotinoids and the contribution of agriculture practices in insecticide resistance selection. The present study aimed to evaluate the resistance profile of *An. gambiae* s.l. to neonicotinoids and other classes of insecticides in two market gardening sites in northern Benin.

## 2. Materials and Methods

### 2.1. Study Sites

**Figure 1:**
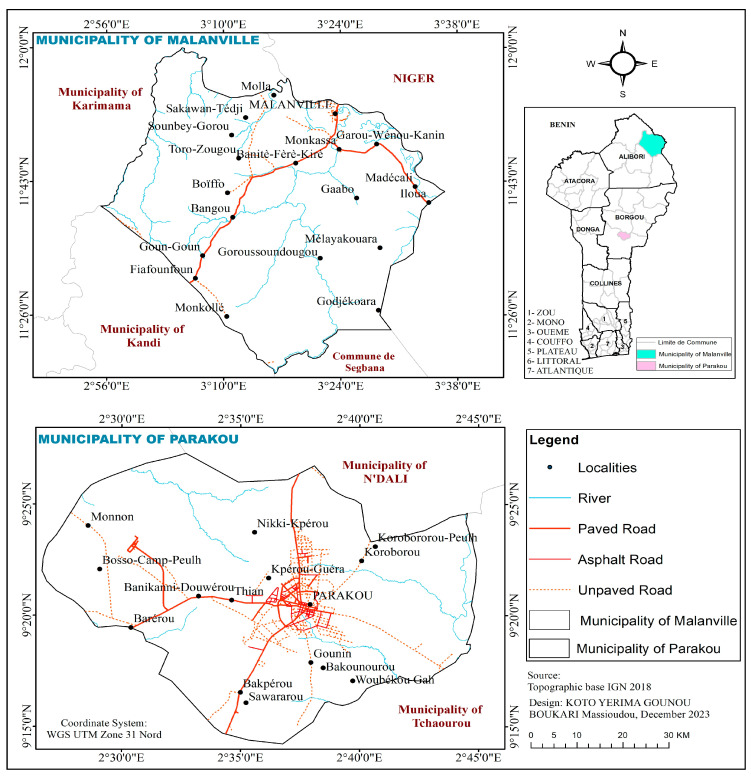
Mapping showing the study sites.

The study was carried out in Malanville and Parakou in northern Benin. Malanville is located between 11°50′38′′ and 11°52′02′′ North latitude and between 3°22′00′′ and 3°24′02′′ East longitude (Figure 1) with a population of 168,006 inhabitants [21]. Its climate is North-Sudanese type marked by a dry season from November to April. The annual average rainfall recorded is 750 mm [9]. The landscape is savannah with grassland and the main agricultural activity is rice cultivation [22] followed by livestock and trading. Rice plots serve as larval habitats for *An. gambiae* s.l. The application of fertilizers and herbicides in agriculture has contributed to the emergence of insecticide resistance in this commune [23].

The district of Parakou (9°20′13′′ N, 2°37′49′′ E) is located at an average altitude of 350 m with a fairly modest relief (Figure 1). It covers an area of 441 km^2^ with a population of 254,254 inhabitants [24]. It is characterized by an annual alternation of a rainy season (May to October) and a dry season (November to April). The lowest temperatures are recorded in December–January. The average annual rainfall is 1200 mm, with a maximum occurring between July and September. These factors are favorable to agriculture. In contrast to Malanville, market gardening (small-scale commercial production of cash crops) is the main activity conducted by inhabitants of Parakou which occur along the commune perimeter and serves as prolific larval habitats for *An. gambiae* s.l. This town has not conducted an IRS campaign so far, but cotton is widely grown with extensive use of insecticides to control agricultural pests [9]. Moreover, people frequently use long-lasting insecticidal nets (LLINs), aerosol sprays, and smoke coils to protect themselves against mosquito bites.

### 2.2. Knowledge, Attitudes, and Practices (KAP) Survey

A socio-anthropological survey was carried out on each of the study sites. Information was collected on the use of agricultural inputs (insecticides, herbicides, and fertilizers), the reasons behind the use of these chemicals, the sources of supply, the period of treatment, the frequent diseases in the community, and the link between the use of chemicals and malaria transmission. Volunteer farmers who gave their consent to the study were interviewed using a well-structured questionnaire approved on 7 September 2023 by the National University of Sciences, Techno logies, Engineering, and Mathematics. They were reassured about the confidentiality of their information. Interviews were conducted in private to reduce bias from other members of the community. Discussions were held with 85 producers, including 40 in Malanville and 45 in Parakou.

### 2.3. Sampling and Rearing of An. gambiae s.l.

*Anopheles gambiae* s.l. larvae were collected by the dipping method [25] with a 350 mL mosquito scoop, in different breeding sites found in vegetable farms in the two prospected localities. For small water bodies where a scoop could not be applied, water was dipped as many times as possible or collected using calibrated pipette. Collection was carried out in Malanville and Parakou between October and November 2023 during five days per locality between 9 a.m. to 5 p.m. *Anopheles* larvae were sorted, rinsed with tap water, and placed in small plastic cups, then transported to the insectary of the International Institute of Tropical Agriculture (IITA) in Benin where they were reared until the emergence of adults. Insectary was maintained under standard conditions of temperature (27 ± 1 °C) humidity (75% ± 5%) and photoperiod (12L:12D). Larvae were reared in plastic trays. They were fed with tetramin baby fish food, whereas adults were fed using 10% honey solution.

### 2.4. Insecticides Susceptibility Tests

#### 2.4.1. WHO Bioassays

Susceptibility tests were carried out with 2050 non-blood-fed female mosquitoes resulting from the emergence of *An. gambiae* s.l. larvae collected in each locality. Emerging females (3 to 5 days) were exposed to insecticides according to WHO protocol [26]. For each insecticide, 4 batches of 25 female mosquitoes were exposed for 1 h to papers impregnated with insecticides.

Lambda-cyhalothrin 0.5%, deltamethrin 0.05%, permethrin 0.75%, bendiocarb 0.1%, DDT 4%, malathion 5%, and pyrimiphos-methyl 0.25% were tested. For each assay, tube tests containing untreated papers were run in parallel as a control. Knockdown was recorded for 1 h and mosquitoes were transferred to observation tubes containing untreated paper, with free access to 10% honey solution. Mortality was recorded 24 h after exposure. The tests were only validated when the mortality observed in the control tubes was less than 5%; therefore, no correction with the Abbott formula was required [26]. At the end of the tests, alive and dead mosquitoes were used for species identification and screening of resistance mechanisms.

#### 2.4.2. CDC Bottle Bioassay

*Anopheles gambiae* s.l. susceptibility to neonicotinoids was screened using Center for Disease Control (CDC) tubes with discriminating doses of acetamiprid (75 μg·L^−1^), imidacloprid (200 μg·L^−1^), and clothianidin (4 μg·L^−1^) [11,27]. Prepared solutions (insecticides mixed with solvent) were stored at 4 °C in the dark for 24 h before use, to optimize solubility [28]. Acetone (Sigma-Aldrich, Steinheim, Germany) was used for acetamiprid and imidacloprid, and acetone + Mero (81% rapeseed oil methyl ester) for clothianidin to prevent its crystallization. The concentration of Mero used was 1.12 μL·mL^−1^ of acetone [27]. Moreover, the tubes were coated with 1 mL of acetamiprid, imidacloprid, and clothianidin (Sigma-Aldrich, Geneve, Switzerland) solution previously prepared. The tubes were protected from light using aluminum foil and allowed to dry overnight for complete evaporation of the solvent before use. The different concentrations were first tested on susceptible *An. gambiae* Kisumu and 100% mortality was recorded. *Anopheles gambiae* Kisumu are fully susceptible strain of *An. gambiae* s.s. species. They are maintained and reared in a separated insectary of AgroEcoHealth platform of IITA-Benin under the standard conditions described above to avoid contamination with a field strain. This strain is used as control to assess the quality of insecticide-impregnated papers before bioassays.

For the tests, four tubes were coated with the target insecticide with two control tubes (one coated with appropriate solvent and the second without solvent). Bioassays were carried out as follows: 4 replicates of 20 to 25 females (3 to 5 days old) were transferred from mosquito cages and gently released into test tubes where they were exposed to the insecticide or into control tubes. The knockdown effect was recorded during one hour of exposure and the mosquitoes were transferred to a paper cup and provided with a 10% sugar solution. Mortality was recorded daily for seven consecutive days under standard laboratory conditions for acetamiprid and imidacloprid assay and just for 24 h for clothianidin assay.

#### 2.4.3. Synergists Assays

To evaluate the different enzymes involved in the resistance of the malaria vectors to neonicotinoids and other classes of insecticides, synergists (Sigma-Aldrich, Taufkirchen, Germany) such as piperonyl butoxide 4% (PBO), di-ethyl maleate 8% (DEM), and s,s,s–tributylphosphorotrithioate 0.25% (DEF) were tested. For each insecticide for which resistance was observed (imidacloprid in Malanville, deltamethrin, lambda-cyhalothrin, permethrin, and DDT), four tubes of 20 to 25 female mosquitoes were firstly exposed to each synergist for one hour before being immediately transferred to the impregnated tubes (WHO tubes or CDC tubes) with the required dose of insecticides as described above. Knockdown was recorded for 1 h and mosquitoes were transferred to observation tubes containing untreated paper. Mortalities were recorded 24 h post-exposure or daily during seven consecutive days for imidacloprid.

### 2.5. Species Identification

To determine the different species of *An. gambiae* complex, the genomic DNA of 343 individuals (alive and dead) and 514 individuals (alive and dead) randomly selected from emerged adults in Malanville and Parakou, respectively, were extracted using the Livak method [29]. The members of *An. gambiae* complex were identified by PCR as described by [30,31].

### 2.6. PCR Detection of the Kdr-L1014F, Kdr-L1014S, N1775Y, and ace-1-G119S

The presence of the *kdr-L1014F, kdr-L1014S, N1775Y* [32,33], and *ace-1-G119S* [34] mutations were screened with TaqMan real-time PCR assay using Agilent Mx3005 qRT-PCR thermocycler (Santa Clara, CA, USA) after genomic DNA extraction was carried out from individual female mosquitoes. TaqMan assay was used to genotype using primers and probes summarized in Table 1. Probes were labelled with two specific fluorophores FAM and HEX, FAM to detect the homozygous resistant genotype, HEX to detect the homozygous susceptible genotype, and both FAM and HEX to detect the heterozygous genotype. The assay was performed on an Agilent MxPro 3005 real-time PCR machine (Santa Clara, CA, USA) with cycling conditions of 95 °C for 10 min, followed by 40 cycles at 95 °C for 15 s and 60 °C for 1 min. FAM and HEX fluorescence was captured at the end of each cycle and genotypes called from endpoint fluorescence using the Agilent MxPro software, version OS v7.10, Control v5.20, Fimware v110.61. Briefly, each reaction was conducted in a total volume of 10 µL that comprise 5 µL Sensimix (Meridian BioScience, Cincinnati, OH, USA), 0.25 µL of 40×Probe Mix coupled to allelic-specific primers, 4.25 µL of ddH_2_0, and 10 μg·mL^−1^ of genomic DNA. At the end of the reaction, genotypes were scored from bi-directional scatter plots of results produced by the Mx3005 v4.10 software.

### 2.7. Data Analysis

Mosquitoes’ resistance to insecticides were defined according to WHO criteria [26]: mortality rate >98% indicates a susceptible population; mortality of 90–98% suspected resistance and mortality; and <90% indicates a resistant population. The Kolmogorov–Smirnov test was used to assess the normality of the distribution. The allele frequencies of the resistance genes *kdr L1014F*, *kdr L1014S*, *N1575Y,* and *ace-1* responsible for resistance to the insecticides tested were calculated and then compared between dead and alive mosquitoes using the Mann–Whitney test in Statistica software version 6. The chi-square test was used to compare data from the KAP survey. Likewise, the results of the bioassays were compared between the insecticides and between the insecticides and their synergists via Mann–Whitney. The difference was considered significant for all analysis at the 5% threshold.

## 3. Results

### 3.1. Knowledge, Attitudes, and Practices (KAP) Surveys

The education level of the farmers interviewed in Malanville was very low (only 10% have been to school), while, in Parakou, 71.11% of respondents had at least some academic training. Among them, 15.63% had a Baccalaureate, 50% reached the secondary school level, and 34.7% reached the primary school level. The socio-demographic data from the KAP surveys were summarized in Table 2.

**Figure 2:**
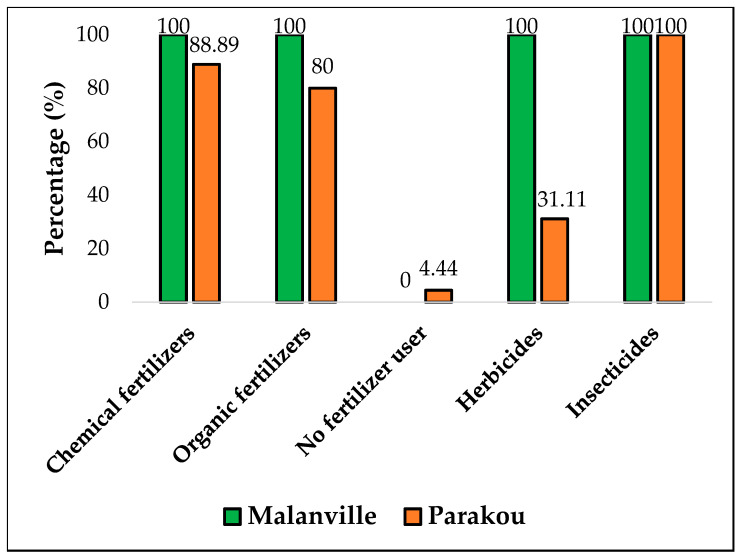
Use of chemicals by market gardeners in Malanville and Parakou.

In Malanville, all the interviewed farmers used chemical fertilizers (100%), herbicides (100%), and pesticides (100%), while, in Parakou, farmers used 95.56%, 31.11%, and 100% of chemical fertilizers, herbicides, and pesticides, respectively (Figure 2). No significant difference was observed on fertilizers and pesticide use between the two localities (Fertilizer: *X*^2^ = 1.821, *df* = 1, and *p* = 0.177). However, a significant difference was observed between these localities on herbicide use (*X*^2^ = 43.374, *df* = 1, and *p* = 4.5204·10^−11^). Farmers (97.5% in Malanville and 93.33% in Parakou) believed that the use of chemicals increase the yield.

The farmers from Malanville obtained the pesticides in the local market or in the Company for the Development of Cotton (SODECO) store, while those of Parakou acquired them in the markets, the shops, or even in the Benin-Seeds sales points.

In the prospected localities, the KAP survey also addressed the use of LLINs by the farmers. In Malanville, 100% of farmers interviewed have at least one mosquito net, and almost all slept under them, while, in Parakou, 95.56% of the population have a mosquito net and 67.44% slept under these nets. The number of people sleeping under mosquito nets in Malanville was significantly higher than those in Parakou (*X*^2^ = 12.645, *df* = 1, *p* = 0.0004).

### 3.2. Resistance Status

#### 3.2.1. Susceptibility Profile of *An. gambiae* s.l. to Neonicotinoids

**Figure 3:**
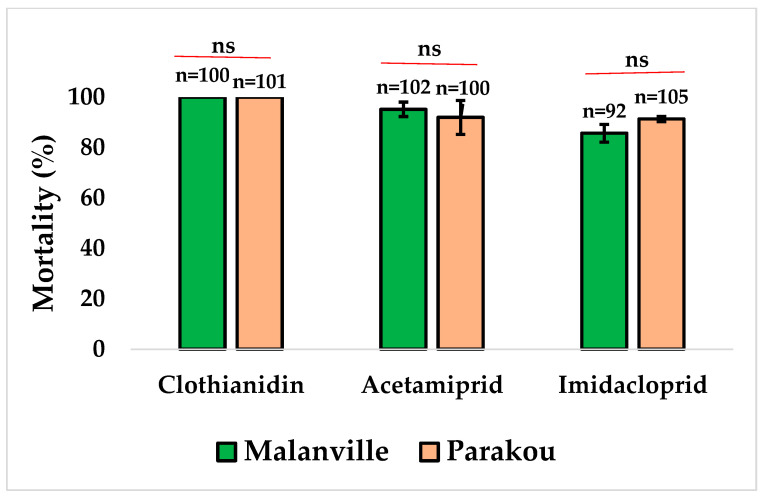
Mortality rate of *An. gambiae* s.l. from Malanville and Parakou exposed to neonicotinoids. Results are average percentage of mortalities from four replicates. N represents number of mosquitoes tested by insecticide. Error bar represents standard deviation, and ns means no significance with Mann–Whitney test.

The bioassays carried out with CDC bottles showed full susceptibility (100% mortality, n = 200) to clothianidin in *An. gambiae* s.l. in both study sites. Suspected resistance to acetamiprid in Malanville (95.19%) and Parakou (92.00%) was recorded (Figure 3). Among the neonicotinoids used in this study, only resistance to imidacloprid (85.71%) was detected in *An. gambiae* s.l. from Malanville and resistance was suspected for the same insecticide in Parakou (92%). Clothianidin significantly showed better results compared to deltamethrin (Mann–Whitney test: Z = 2.46, and *p* = 0.014) and permethrin (Mann–Whitney test: Z = 2.477, and *p* = 0.013) in Malanville. Similar results were recorded in Parakou for deltamethrin (Mann–Whitney test: Z = 2.460, and *p* = 0.014) and permethrin (Mann–Whitney test: Z = 2.46, and *p* = 0.014).

#### 3.2.2. Susceptibility Profile of *An. gambiae* s.l. to Pyrethroids, Organophosphates, and DDT

**Figure 4:**
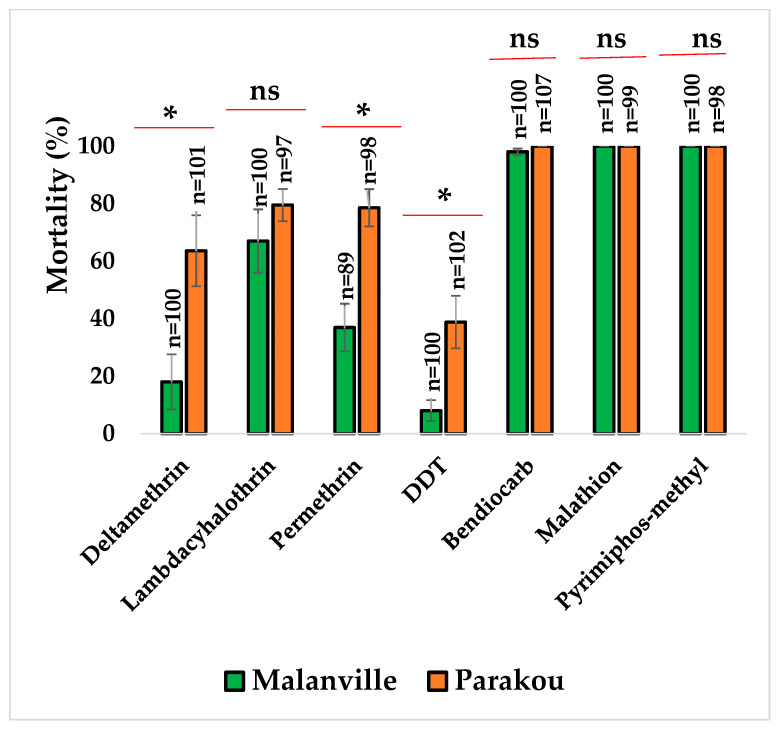
Insecticide resistance status of *An. gambiae* s.l. from Malanville and Parakou. Results are average percentage of mortalities from four replicates. N represents number of mosquitoes tested by insecticide. Error bars represent standard deviation. ns means no significance with Mann–Whitney test. * means a significant difference between the insecticide (Mann–Whitney test).

The resistance of *An. gambiae* s.l. to lambda-cyhalothrin, deltamethrin, permethrin, and DDT was observed in Malanville and Parakou (Figure 4). Bioassays carried out on *An. gambiae* s.l. collected from the two study sites showed a susceptibility to bendiocarb, malathion, and pyrimiphos-methyl (Figure 4).

### 3.3. Synergists Effects

#### 3.3.1. Synergist Tests with Imidacloprid

**Figure 5:**
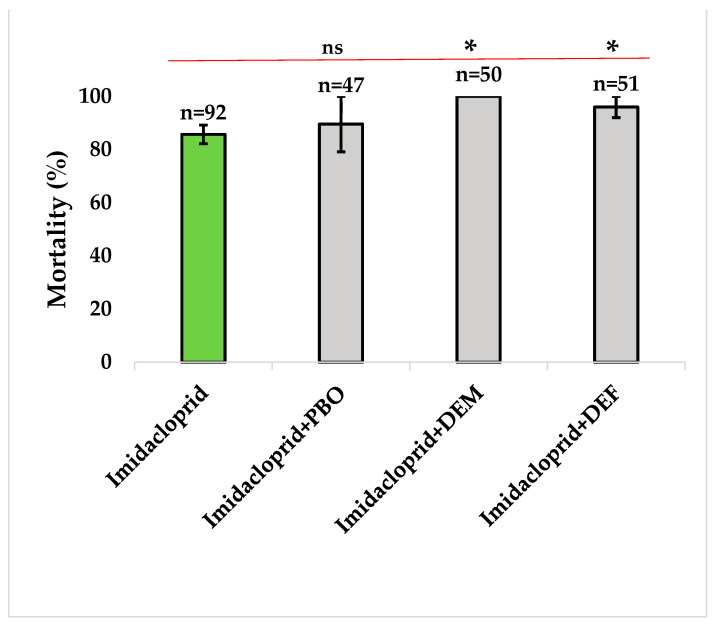
Bioassay with synergists combined with imidacloprid in *An. gambiae* s.l. collected from Malanville. Results are average percentage of mortalities from four replicates. n represent number of mosquitoes tested by insecticide. Error bar represent standard deviation. The stars (*) represent the significance level and ns means no significance. Green sticks represent insecticides and grey sticks represent synergists.

When imidacloprid was pre-exposed with synergists (PBO, DEF, or DEM), the mortality rate increased compared to imidacloprid alone. The synergists DEM (Z = −2.460, *p* = 0.014) and DEF (Z = −2.337, *p* = 0.019) in Malanville increased mortality, showing the implication of glutathione-s-transferases and esterases in the observed resistance (Figure 5).

#### 3.3.2. Synergist Tests with Other Classes of Insecticides

Biossays using synergists significantly increased the mortality rates of pyrethroids and DDT. The pre-exposure of mosquitoes to PBO, DEM, and DEF significantly increased the mortality rates of lambda-cyhalothrin (PBO: Z = −2.477, *p* = 0.013; DEM: Z = −2.477, *p* = 0.013; DEF: Z = −2.477, *p* = 0.013), permethrin (PBO: Z = −2.351, *p* = 0.019; DEM: Z = −2.351, *p* = 0.019), and deltamethrin (PBO: Z= −2.4604; *p* = 0.014; DEM: Z = −2.337, *p* = 0.019; DEF: Z = −2.337, *p* = 0.019) in Malanville. Similar results were recorded in Parakou for lambdacyhalothrin (PBO: Z = −2.460, *p* = 0.014; DEM: Z = −2.460, *p* = 0.0134), deltamethrin (PBO: Z = −2.460, *p* = 0.014; DEM: Z = −2.337, *p* = 0.019; DEF: Z = −2.460, *p* = 0.014), and permethrin (Z = −2.460, *p* = 0.014).

**Figure 6:**
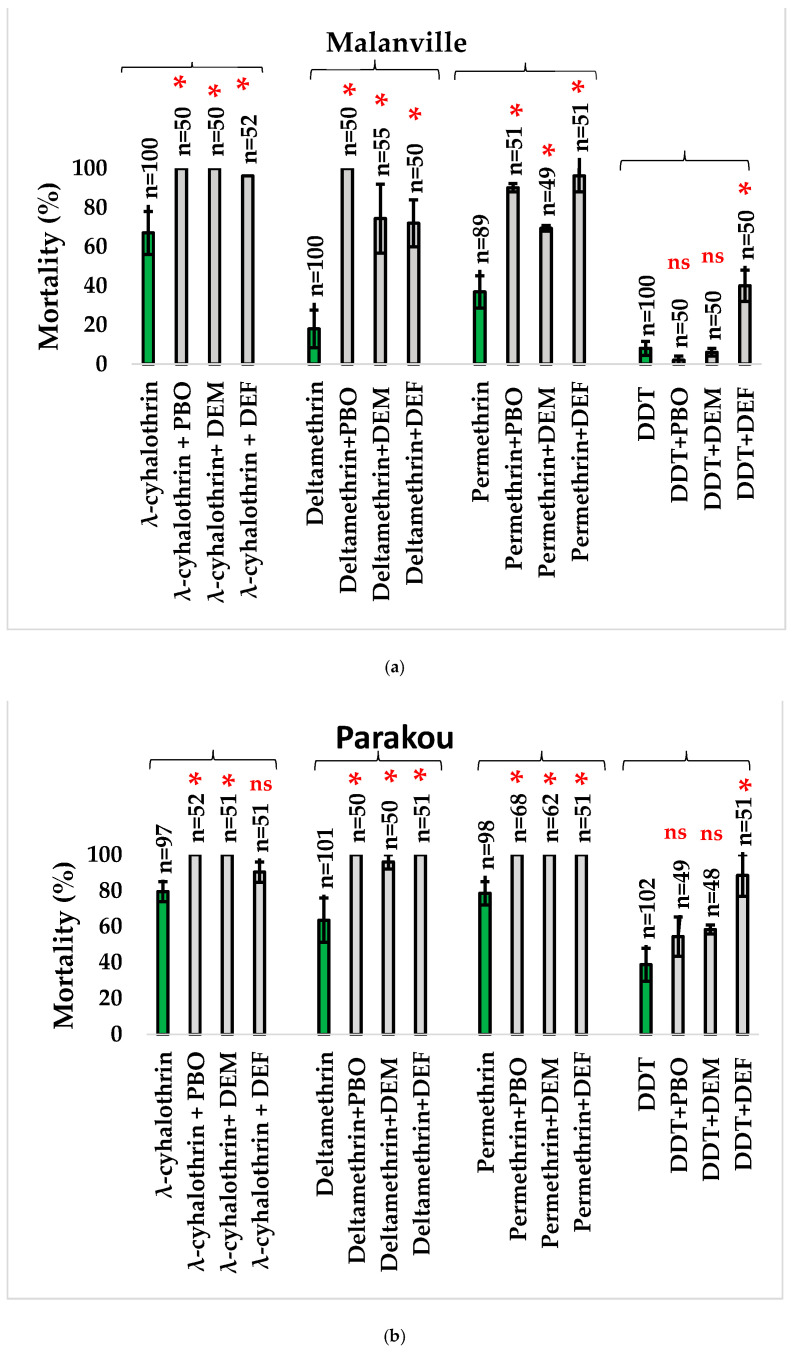
WHO bioassays showing the efficacy of insecticide in Malanville (**a**) and Parakou (**b**) with and without pre-exposure to the synergists (error bar represents standard deviations). n = number of mosquitoes tested by insecticide. The stars (*) represent significance level and ns means no significance. Green sticks represent insecticides and grey sticks represent synergists.

For DDT, only the pre-exposure to DEF significantly increased the mortality rates (Z = −2.337, *p* = 0.019) in Parakou and Malanville (Z= −2.337, *p* = 0.019). The results from bioassays with synergists suggested that oxidases were more involved in the resistance of *An. gambiae* s.l. to pyrethroids while esterases were better involved in the resistance of *An. gambiae* s.l. to DDT (Figure 6).

### 3.4. Species Identification of An. gambiae s.l.

From the 857 females analyzed, 529 (61.73%) were *An. gambiae*, 315 (36.76%) *An. coluzzii*, 6 (0.7%) *An. Arabiensis,* and 7 (0.82%) were hybrids.

Out of the 343 mosquitoes collected in Malanville, 6 (1.75%) were *An. arabiensis*, 7 (2.04%) were hybrids, 15 (4.37%) were *An. gambiae*, and 315 (91.84%) were *An. coluzzii,* while, in Parakou, all mosquitoes tested (514) belong to *An. gambiae*.

### 3.5. Detection of Resistance Genes

Overall, the allelic frequencies of *kdr-L1014S, N1575Y*, and *ace-1* were very low in both dead and alive mosquitoes in Malanville. The allelic frequencies of *kdr-L1014F* was high and similar among alive and dead mosquitoes from Malanville and Parakou, suggesting that this mutation was playing no or little role in the observed resistance. Allelic frequencies of *kdr L1014F*, *kdr L1014S, N1575Y*, and *ace-1 G119S* are summarized in Table 3.

## 4. Discussion

This work aims to raise awareness of the widespread resistance of *An. gambiae* s.l. to pyrethroid and organochlorine insecticides and demonstrate the susceptibility to carbamates, organophosphates, and clothianidin in order to highlight the importance for better insecticide management. Information collected during interviews with farmers and the observations made in the two study sites confirmed a common use of fertilizers and insecticides in the vegetable farm. The development of vegetable farming requires the intensive use of pesticides including insecticides belonging to the main classes recommended for vector control in public health: pyrethroids, organophosphates, and organochlorines. In this study, we assessed cross-resistance in *An. gambiae* s.l. from vegetable farming and explored the molecular mechanisms involved.

In Benin, several studies in line with this work have reported that malaria vectors have been resistant to insecticides such as pyrethroids, organochlorine, and carbamate classes for several decades [5,8,9,13,23,35,36,37,38,39,40,41]. In addition to the LLINs and IRS, agricultural practices are pointed out as contributing to gene selection pressure and a rise in insecticide resistance intensity [4]. Considering that the breeding sites in agricultural areas are constituted of the run-off water containing agricultural chemicals, the link between the resistance of these vectors and agricultural practices has well been established [10,35,42,43].

Bioassays carried out in this study revealed that *An. gambiae* s.l. was resistant to permethrin, deltamethrin, lambda-cyhalothrin, and DDT in Malanville and Parakou. These results confirmed the information recorded from the KAP survey about the use of these insecticides to control plant pests. The association between the agricultural usage of pesticides and the development of insecticide resistance in *Anopheles* mosquitoes is widely documented and many studies have hypothesized that pesticide residues contaminate mosquito breeding sites and contribute to the resistance selection to insecticides in mosquito population [43,44]. The observed resistance was higher in Malanville compared to Parakou, confirming the information recorded from the KAP survey about the use of these chemicals to control plant pests and weeds, and to fertilize soils. Indeed, the information provided by the farmers of these two localities revealed that farmers in Malanville use more chemical fertilizers and herbicides as this locality is a cotton- and rice-growing area. Susceptibility tests carried out, with neonicotinoids, showed that mosquitoes were resistant to imidacloprid in Malanville, whereas a suspected resistance to acetamiprid was pointed out in both study sites. Mortality increases gradually from day 1 to day 7 after exposure to acetamiprid and imidacloprid, confirming the slow action of neonicotinoids on the nervous system of the target insects, as previously reported by several studies [45,46,47]. Acetamiprid and imidacloprid were used in agriculture in Benin and especially in the fight against plant pests and other domestic insects [20,48,49]. Interestingly, full susceptibility to clothianidin was observed in both localities. Indeed, all the females of *An. gambiae* s.l. exposed to clothianidin dissolved in acetone + Mero oil died after 24 h comparing to the results obtained by Zoungbédji et al. [13] in rice-growing environments of the same locality but using water as solvent, suggesting that Mero occurred in the decrystallization and facilitate clothianidin absorption to become more effective [27,50]. Neonicotinoids appeared to be effective in the surveyed sites; this could be explained by the fact that the insecticides used by farmers likely contain few neonicotinoids. There is a need of sensitization, farmers training on the use of pesticides, and good agricultural practices for the sustainability of vectors’ control tools. Regarding the distribution of malaria vector species, all mosquitoes collected from Parakou were *An. gambiae,* while, in Malanville, there was a predominance of *An. coluzzii* (91.84%). Previous studies revealed the predominance of *An. coluzzii* in Malanville associated with *An. arabiensis* and/or *An. coluzzii* in the same locality [51]. Significant spatial variation in the prevalence of the *Anopheline* species between the northern districts of the country was shown by other studies [5,7,12,51,52].

Genotyping of the knockdown resistance mutations revealed the presence of *kdr L1014F* at the moderate frequencies with no significant difference in allelic frequencies between dead and alive mosquitoes, suggesting that this mutation is not protecting the mosquitoes against insecticides. Hence, metabolic resistance could be the main resistance mechanism used by *An. gambiae* s.l. to fight against pyrethroids insecticides as shown by synergist assays. These assays indicated how often detoxification enzymes, notably monoxidases and gluthation S-transferases, are involved in the resistance of these vectors to pyrethroids [6,53,54,55] The allelic frequency of the Ace-1 mutation was poorly represented in the analyzed mosquitoes which could be explained by the susceptibility obtained with carbamates and organophosphates. These results highlighted the ability of the mosquitoes in using different mechanisms to resist to different insecticides. The high levels of susceptibility of field populations to clothianidin indicate a lack of cross-resistance with known pyrethroid insecticides. The target site of this insecticide is different from that of pyrethroids and the lack of cross-resistance indicates that enzymes involved in the metabolic detoxification of pyrethroids do not affect clothianidin [56]. However, the reduction in resistance to imidacloprid after the pre-exposure of mosquitoes to DEM and the improvement in sensitivity with DEF indicate the involvement of glutathione S-transferases and esterases in the resistance of malaria vectors to this insecticide, as previous studies have already reported on the role of detoxification enzymes in the resistance mechanisms of *Anopheles* to neonicotinoids [28,45,57].

## 5. Conclusions

The results of this study confirmed the widespread resistance of *An. gambiae* s.l. to pyrethroid and organochlorine insecticides. However, a susceptibility of these vectors to carbamates, organophosphates, and clothianidin was recorded, showing that this insecticide can be explored in improving vector control tools. Furthermore, the suspected and/or resistance of these mosquitoes to acetamiprid and imidacloprid in the surveyed sites requires raising awareness among populations on agricultural practices to maintain the effectiveness of clothianidin. Finally, the different levels of resistance to neonicotinoids observed in the localities surveyed suggest that *An. gambiae* s.l. is using different mechanisms against this class of insecticide. These observations call for further investigation in order to improve the implementation and management of future control programs against this species and other *Anopheles* mosquitoes locally involved in malaria transmission.

## Figures and Tables

**Table 1:** Taqman-PCR primers for mutations analysis.

Primers	Sequences
kdr-Forward	5′-CATTTTTCTTGGCCACTGTAGTGAT-3′
kdr-Reverse	5′-CGATCTTGGTCCATGTTAATTTGCA-3′
probe WT	5′-CTTACGACTAAATTTC-3′
probes kdr-W	5′-ACGACAAAATTTC-3′
primer G119-R1	5′-CGGTGGTCGTACACGTCCAGGGT-3′
G119S-F1	5′-GCGGGCAGGGCGGCGGGGGCGGGGCCCTGTGGATCTTCGGCGGCG-3′
primer G119R-F1	5′-GCGGGCCTGTGGATCTTCGGCGGCA-3′

All primers were obtained from Applied Biosystems (Waltham, MA, USA).

**Table 2:** Socio-demographics data of KAP survey (Supplementary Material).

Parameters	Characteristics	Malanville n (%)	Parakou n (%)
Age	less than 20	0 (0.00)	5 (11.11)
	20 to 30	11 (27.50)	24 (53.33)
	31 to 40	14 (35.00)	9 (20.00)
	41 to 50	5 (12.50)	3 (6.67)
	51 to 60	6 (15.00)	3 (6.67)
	more than 60	4 (10.00)	1 (2.22)
Sex	Male	40 (100.00)	45 (100.00)
	Female	0 (0.00)	0 (0.00)
Main activity	Market gardening	15 (37.50)	43 (95.56)
	Agriculture	25 (62.50)	2 (4.44)
Education	No formal schooling	36 (90.00)	13 (28.89)
	Primary	0 (0.00)	11 (24.44)
	Secondary	4 (10.00)	16 (35.56)
	University	0 (0.00)	5 (11.11)
Year from which these pesticides are used	0 to 10	21 (52.50)	35 (77.78)
	11 to 20	8 (20.00)	6 (13.33)
	More than 20	11 (27.50)	4 (4.44)
Recognition of the effectiveness of pesticides	State of health of the plant	31 (77.50)	40 (88.89)
	Low number of pests	0 (0.00)	3 (6.67)
	Both	9 (22.50)	2 (4.44)
Number of persons surveyed by site		40	45

For this study, we did not specify the gender; no women were found in sampling sites.

**Table 3:** Allelic frequencies of *kdr L1014F*, *kdr L1014S*, *N1575Y*, and *ace-1 G119S* in dead and alive mosquitoes exposed to insecticides.

Localities	Mutations	Alive					Dead					
		N	RR	RS	SS	f(R)	N	RR	RS	SS	f(R)	*p* Value (Z = Value of the Normality)
Malanville	*kdr West*	60	22	19	19	0.53	60	12	31	17	0.46	0.26 (Z = 1.107)
	*kdr East*	60	0	7	53	0.06	60	0	0	60	0	0.33 (Z = −0.857)
	*ace-1*	60	0	1	59	0.01	60	2	4	54	0.07	0.04 (Z = −2.087)
	*N1575Y*	60	0	4	56	0.03	60	0	7	53	0.06	0.07 (Z = −1.771)
Parakou	*kdr West*	60	33	9	18	0.63	60	27	2	31	0.47	0.08 (Z = 1.732)
	*kdr East*	60	0	22	38	0.18	60	0	15	45	0.12	0.93 (Z = −0.080)
	*ace-1*	60	0	0	60	0	60	1	0	59	0.02	0.27 (Z = −1.095)
	*N1575Y*	60	0	0	60	0	60	0	0	60	0	-

N = total number of mosquitoes tested, RR = resistant homozygotes, RS = heterozygotes, SS = susceptibility homozygotes, f(R) = mutation frequency, *p* value = significance level; Mann–Whitney test at 95% confidence interval has been used.

## Data Availability

All data related to the research are included in this article.

## Supplementary Materials

The following supporting information can be downloaded at: https://www.mdpi.com/article/10.3390/tropicalmed9120305/s1. KAP survey data base.
